# Attachment of *Salmonella enterica* on Mangoes and Survival Under Conditions Simulating Commercial Mango Packing House and Importer Facility

**DOI:** 10.3389/fmicb.2018.01519

**Published:** 2018-07-10

**Authors:** Elza N. Mathew, Muhammed S. Muyyarikkandy, Deepa Kuttappan, Mary Anne Amalaradjou

**Affiliations:** Department of Animal Science, University of Connecticut, Storrs, CT, United States

**Keywords:** *Salmonella*, attachment, fructoplane, survival, mangoes, mango packing house, importer facility

## Abstract

Consumption of raw mangoes has led to multiple *Salmonella-*associated foodborne outbreaks in the United States. Although several studies have investigated the epiphytic fitness of *Salmonella* on fresh produce, there is sparse information available on the survival of *Salmonella* on mangoes under commercial handling and storage conditions. Hence, the objective of the study was to evaluate the survival of *Salmonella* on mangoes under ambient conditions simulating the mango packing house and importer facility. Further, the ability of the pathogen to adhere and attach on to the mango fructoplane was also investigated. For the attachment assays, mango skin sections were inoculated with fifty microliters of *S.* Newport suspension (6.5 log CFU/skin section) and minimum time required for adhesion and attachment were recorded. With the survival assays, unwaxed mangoes were spot inoculated with the *Salmonella* cocktail to establish approximately 4 and 6.5 log CFU/mango. The fruits were then subjected to different storage regimens simulating fruit unloading, waxing, and storage at the packing house and ripening and storage at the importer facility. Results of our study reveal that *Salmonella* was able to adhere on to the fructoplane immediately after contact. Further, formation of attachment structures was seen as early as 2 min following inoculation. With the survival assays, irrespective of the inoculum levels, no significant increase or decrease in pathogen population was observed when fruit were stored either at ambient (29–32°C and RH 85–95%, for 48 h), ripening (20–22°C and RH 90–95% for 9 days) or refrigerated storage (10–15°C and 85–95% for 24–48 h) conditions. Therefore, once contaminated, mangoes could serve as potential vehicles in the transmission of *Salmonella* along the post-harvest environment. Hence development and adoption of effective food safety measures are warranted to promote the microbiological safety of mangoes.

## Introduction

Although consumption of fresh produce is considered safe, recent produce-associated illnesses have highlighted their potential for transmission of foodborne pathogens (Luo et al., 2012). Among the different foodborne pathogens, *Salmonella enterica* has been implicated in several produce-related outbreaks (Collignon and Korsten, 2010; Reddy et al., 2016). In effect, recent source attribution studies estimate that fruits and vegetables were implicated in about 50% of *Salmonella* outbreaks (Centers for Disease Control and Prevention [CDC], 2015). Toward this, foodborne salmonellosis has been associated with the consumption of contaminated mangoes, blueberries, watermelon, cantaloupe, grapes, tomatoes, cucumbers, and sprouts (Centers for Disease Control and Prevention, National Center for Emerging, and Zoonotic Infectious Agents [CDC NCZEID], 2017). With specific reference to mangoes, there have been seven confirmed *Salmonella* outbreaks in the United States between 1998 and 2014 (Centers for Disease Control and Prevention, National Center for Emerging, and Zoonotic Infectious Agents [CDC NCZEID], 2017). In all of these cases, the outbreaks were traced back to raw mangoes (Strawn and Danyluk, 2010).

The presence of a pathogen on a fruit’s surface indicates that product contamination potentially occurred along the production continuum. In this regard, mangoes can get contaminated within the pre and post-harvest environments (Fatica and Schneider, 2011; Penteado, 2017). In the orchards, mango trees are cultivated and fruit are harvested in their natural environment where they are exposed to a variety of contamination sources including soil, irrigation water, manure, and animals in or near the field (Fatica and Schneider, 2011; Huff et al., 2012; Gautam et al., 2014). This of particular significance with pathogens such as *Salmonella* since they normally reside in the intestinal tract of animals and can therefore gain entry into the pre-harvest environment through contaminated animal feces (Fatica and Schneider, 2011; Tomas-Callejas et al., 2011). In addition to soiling produce in the field, pathogen transmission and contamination can occur during post-harvest handling, processing, and distribution. Produce contamination is further complicated by intensive farming practices, large-scale distribution of the produce and globalization of food supply (Hanning et al., 2009). Moreover, eliminating pathogens from produce such as mangoes is challenging since these are consumed as raw, fresh commodity. Therefore contamination could occur at any point from the farm and packing house up to the point of handling and consumption (Collignon and Korsten, 2010). Hence, in addition to incorporation of good agricultural practices (GAPs), the produce industry relies on good management practices employed along the supply chain to improve the microbial safety of fresh produce (Goodburn and Wallace, 2013).

For the successful transmission of a pathogen through fresh produce, the initial process of adhesion and attachment on the plant surface is highly critical (Tan et al., 2016; Fornefeld et al., 2017). Adhesion refers to the reversible adsorption of bacteria to the substrate surface, which is the phylloplane or fructoplane in case of produce (Garrett et al., 2008). At this stage, application of a strong repulsive force can dislodge the adhered bacterial cells. However, a fraction of the reversibly adhered cells remain immobilized and become irreversibly adsorbed or attached. The process of attachment is mediated by the production of bacterial appendages (flagella, fimbriae, and pili) and exopolysaccharide (Garrett et al., 2008; Collignon and Korsten, 2010; Tan et al., 2016). Once attached, bacterial cells can then replicate and are usually found incorporated in phylloplane biofilms (Fett, 2000; Monier and Lindow, 2005). Within these biofilms, the pathogen is protected from environmental fluctuations, thereby promoting its survival on fresh produce (Marshall, 1992; Morris and Monier, 2003; Collignon and Korsten, 2010).

Survival of pathogens on produce is influenced by ambient conditions such as those encountered at the packing house and during transport including temperature and relative humidity (Doyle and Erickson, 2008; Collignon and Korsten, 2010; Francis et al., 2012; Penteado, 2017). Additionally, post-harvest practices including waxing can influence pathogen persistence and contamination on produce (Francis et al., 2012). In the light of the multiple *Salmonella* outbreaks, it is clear that mangoes can serve as reservoirs of foodborne pathogens. Therefore, there is a need to develop and implement preventive measures to control pathogen contamination on mangoes. However, in order to develop effective food safety practices, it is essential to determine pathogen behavior on mangoes under mango handling, storage, and distribution conditions. Consequently, the goal of this study was to understand the potential of *S. enterica* to adhere and survive on mangoes under conditions simulating a commercial packing house and importer facility. **Table 1** presents the different ambient conditions and practices employed at a commercial packing house and importer facility. The set temperatures, relative humidity, and length of storage were adapted from the National Mango Board (NMB) mango post-harvest best management practices manual and the NMB mango handling and ripening protocol (National Mango Board [NMB], 2014a,b).

**Table 1:** Ambient and storage conditions employed at a commercial mango packing house and importer facility.

Stage	Holding temperature (°C)	Relative humidity (%)	Length of holding
Unloading and staging^∗^	29–32	85–95	48 h
Waxing, storage, and distribution^∗^	10 (Tommy Atkins) 12 (Ataulfo)	85–95	48 h
Ripening^†^	20–22	90–95	9 days
Storage (post-ripening) and distribution^†^	12–15	90–95	24 h

*Adapted from the Mango postharvest best management practices manual (National Mango Board [NMB], 2014b) and Mango handling and ripening protocol (National Mango Board [NMB], 2014a).*

*^∗^At the mango packing house. ^†^At the importer facility.*

## Materials and Methods

### Bacterial Cultures

One isolate each from six different serovars of *S. enterica* (*S*. Montevideo, *S*. Poona, *S.* Newport, *S*. Baildon, *S*. Braenderup, and *S*. Saintpaul – tomato outbreak isolates) were used in the study. These isolates were kindly provided by Dr. Venkitanarayanan (Department of Animal Science, University of Connecticut, Storrs, CT, United States). Since *S.* Newport and *S.* Branderup have been previously associated with *Salmonella* outbreaks associated with mangoes, although isolated from tomatoes, similar serovars were employed in the study. Further, this study was done as a follow up to a recent study investigating the efficacy of commercially employed wash water disinfectants in controlling Salmonella in wash water and on mangoes (Mathew et al., 2018). Therefore, in order to understand the behavior of these isolates on mangoes during the post-harvest handling and storage of mangoes, the same bacterial cultures were utilized in the present study. All the six isolates were induced for resistance to nalidixic acid (NA; Sigma–Aldrich, St. Louis, MO, United States; 50 μg/ml) to facilitate selective enumeration of the inoculated pathogens (Harris et al., 2001).

### Preparation of Inoculum

Each isolate was cultured separately in 10 ml of sterile tryptic soy broth (TSB, BD Difco, Becton, Dickson and Company, Sparks, MD, United States) containing NA (50 μg/ml) at 37°C for 24 h with agitation (100 rpm). Cultures were then transferred for 24 h period onto xylose deoxycholate (XLD; Difco) agar plates containing NA (50 μg/ml; XLDN) to produce a bacterial lawn. To prepare the inoculum, sterile buffered peptone water (BPW, Difco) was added to each plate and bacterial cells were loosened with a sterile spreader. Approximate bacterial count in each culture was determined spectrophotometrically. Equal volumes containing approximately equal populations from each of the six isolates were combined to make the pathogen cocktail. The bacterial count in each culture and the cocktail was determined by dilution and plating on XLDN. Appropriate dilutions of the cocktail in BPW was used to obtain the desired level of inoculum (6.5 or 4 log CFU/mango). A high inoculum level was used to enable measurement of several log reductions in pathogens counts during the study (Beuchat et al., 2001). Additionally, this study incorporated a low level of inoculum (4 log CFU/mango) in order to simulate low levels of pathogen contamination that are likely to occur under normal processing, storage and distribution conditions (Behrsing et al., 2003).

### Mangoes

Unripened, unwaxed mangoes (var. Tommy Atkins and Ataulfo) were used in the study. Tommy Atkins, a large, round mango, is the most widely grown commercial variety coming into the United States. It is also shown to possess long shelf life and tolerance to handling and transportation (Araiza et al., 2005). Ataulfo mangoes were used to simulate the effect of different processing and storage conditions on small sized flat mango varieties (National Mango Board [NMB], 2014b). Upon receipt, fruits were visually inspected for defects (bruises, moldy growth, breaks in peel) and any defective mango was discarded. All the fruits were maintained at 4°C until use. A day before the experiment, the required number of fruits were transferred to room temperature (21°C) for tempering prior to use (Penteado et al., 2004).

### Adhesion and Attachment of *Salmonella* on Mangoes

#### Enumerating Adhered *Salmonella* on Mango Surface

Given the high incidence of *S.* Newport with produce-related outbreaks and its implication in previous mango-associated Salmonellosis, attachment assays, and electron microscopy were performed using this serotype (Sivapalasingam et al., 2003; Reddy et al., 2016). For assessment of *Salmonella* attachment on mango surface, skin sections (5 mm × 5 mm) were spot inoculated with 6.5 log CFU of *S.* Newport (50 μl) and held for 0, 30, and 60 s, 2 min, and 1 h at room temperature The inoculum was aspirated at the respective time intervals and subsequently rinsed with double the volume of inoculum ca. 100 μl of sterile distilled water was applied to the inoculation site and rinsed by pipetting the solution up and down for five times. rinsate was then aspirated and discarded, and the rinsing process was repeated two times (Collignon and Korsten, 2010). The inoculated skin section were then transferred to 10 ml BPW and vortexed for 2 min followed by microbiological analysis. Ten sections (from five mangoes) were sampled at each time point and the entire experiment was repeated three times.

#### Scanning Electron Microscopy (SEM)

For SEM studies, mangoes were dipped in 70% ethanol for 30 s to remove background microflora, mangoes were then peeled and skin sections (5 mm × 5 mm) were cut out using a sterile knife. Spot inoculation was then performed by placing 50 μl (6.5 log CFU) of *S.* Newport on to the mango skin section at room temperature and aspirated at 0, 30, and 60 s, 2 min, and 1 h. The inoculated sections were subsequently rinsed with 100 μl of sterile distilled water. The rinsate was then aspirated and discarded, and the rinsing process was repeated. The inoculated sections were then processed for SEM (Collignon and Korsten, 2010). Briefly, skin sections were fixed in 5% glutaraldehyde–PBS buffer (1:1, v/v) for 1 h (25°C). The sections were then washed for 10 min in PBS buffer. The dehydration step consisted of serial treatments in ethanol, consisting of 30, 50, 70, 80, and 95% ethanol for 10 min each and three treatments of 100% ethanol for 15 min each. The samples were then transferred to a critical point drier (Critical Point Dryer 931GL, Tousimis, Rockville, MD, United States) for total dehydration. The samples were finally gold sputter coated and images were recorded using a SEM (Nova NanoSEM 450, FEI, Hillsboro, OR, United States; Fernandes et al., 2014). Uninoculated skin sections served as the control fructoplane.

### Survival of *Salmonella* on Mangoes Under Simulated Fruit Handling, Waxing, Ripening, and Storage Cconditions

#### Spot Inoculation

Mangoes were spot inoculated with 6.5 log or 4 log CFU/mango by placing 50 μl of the *Salmonella* cocktail around the stem end. In order to prevent the inoculum from running off the side of the mango, the inoculum was applied in small approximately equal volumes to 10 different locations (Lang et al., 2004; Baskaran et al., 2013). After inoculation, mangoes were held at room temperature for 24 h before being subjected to storage (Wei et al., 1995; Kenney and Beuchat, 2002; Sheng et al., 2017). Staggered inoculation of the mangoes was performed to maintain consistent drying time for all mangoes under each objective (Parnell and Harris, 2003). Before each experiment, 12 mangoes were sampled immediately following inoculation and after drying (24 h post-inoculation) to ascertain starting pathogen load and inoculum uniformity on the fruits (Sheng et al., 2017).

#### Unloading and Staging Conditions at the Packing House [Temperature – 29–32°C (Ambient Temperature), RH – 85–95%, Length of Storage (2–48 h)]

Following inoculation and drying, mangoes were placed in unsealed sterile polycarbonate containers (8-3/4 × 8-5/16 × 8-3/4 in; Fisher Scientific, Waltham, MA, United States) and stored at 29–32°C (31 ± 1°C), RH 85–95% (90 ± 3), for a time period of 2–48 h to simulate mango handling during transportation to and staging at the mango packing facility (National Mango Board [NMB], 2014b; **Table 1**). In the present study, mangoes were held or stored in unsealed containers to simulate the use of plastic crates during fruit holding and transport in the mango industry (Esguerra and Rolle, 2018). Relative humidity was monitored throughout the experiment using digital humidity/temperature/dew point meter (Traceable^TM^, Fisher Scientific, Hampton, NH, United States). At designated times during storage (0, 2, 12, 24, and 48 h) mangoes were sampled for microbiological analysis.

#### Waxing, Storage, and Distribution [10°C (Tommy Atkins) or 12°C (Ataulfo), RH 85–95% for 2–48 h] Conditions at the Packing House

Following inoculation and drying, mangoes were sprayed with a Carnauba-based wax preparation using a gravity-feed dual action air-nozzle sprayer (Carnauba Gold II, Pace International LLC, Wapato, WA, United States). Carnauba-based wax was used since it is the most commonly used wax in the mango industry (National Mango Board [NMB], 2014b). Each mango was sprayed with one pull each to the stem and calyx ends and three pulls to coat the rest of the mango at 29–32°C (30 ± 1°C, Kenney and Beuchat, 2002). The one to three pull using a gravity sprayer was performed to ensure uniform application of wax over the fruit surface. Following waxing, the mangoes were dried at ambient temperature (30 ± 1°C) for 2 h. A subset of mangoes were sampled to ascertain pathogen load on mangoes prior to storage. Then, mangoes were placed in sterile containers and stored at 10°C (var. Tommy Atkins) or 12°C (var. Ataulfo), RH 85–95% (90 ± 3), for a time period of 2–48 h to simulate mango storage at and transportation from the mango packing facility (National Mango Board [NMB], 2014b; **Table 1**). At designated times during storage (0, 2, 12, 24, and 48 h) mangoes were sampled for microbiological analysis.

#### Fruit Ripening (20–22°C, RH 90–95% for 9 Days), Storage and Distribution (12–15°C, RH 90–95% for 2–24 h) Conditions at the Importer Facility

Following inoculation and drying, waxed mangoes were placed in sterile containers and stored at 20–22°C (21 ± 1°C), RH 90–95% (92 ± 3), for a time period of 9 days for mango ripening (National Mango Board [NMB], 2014a). At designated times during storage (0, 1, 3, 5, 7, and 9 days) mangoes were sampled for microbiological analysis. Following ripening, mangoes were then stored at 12–15°C (14 ± 1°C), RH 90–95% (92 ± 3), for a time period of 2–24 h in to simulate mango storage at and transportation from the importer facility (National Mango Board [NMB], 2014a; **Table 1**). At designated times during storage (0, 2, 12, and 24 h) mangoes were sampled for microbiological analysis.

### Microbiological Analysis

At each sampling time, mangoes (*n* = 4) were individually transferred to sterile stomacher bags containing 200 ml of BPW. Each mango was hand rubbed from outside its bag for 2 min, and BPW from each sample was concentrated by centrifugation and serially diluted in 0.1% BPW and appropriate dilutions were surface plated on XLDN agar plates (Harris et al., 2001). In addition to enumeration, BPW samples were enriched in Rappaport–Vassiliadis broth R10 (RVB, Difco) and incubated at 43°C for 16–24 h (Parnell et al., 2005). When counts for the respective samples were negative by direct plating, enrichment broths were streaked on XLDN and incubated at 37°C for 48 h. Presumptive colonies isolated on XLDN agar plates were confirmed as *S. enterica* by agglutination assays (*Salmonella* latex agglutination test, Microgen Bioproducts Ltd, Surrey, United Kingdom).

### Statistical Analysis

Four mangoes were sampled at each sampling time and three independent trials were conducted for each experiment. Pooled samples were averaged and the data was analyzed using the mixed procedure of SAS (Statistical Analysis Software) ver. 9.2. Differences among the means were detected at *p* < 0.05 using the Fisher’s least significance difference test. Independent experiments following the above mentioned procedures were conducted to determine the effect of mango packing house environment and distribution conditions on *Salmonella* persistence using high and low inoculum on Tommy Atkins and Ataulfo mangoes.

## Results

Results of our study did not demonstrate any significant difference in *Salmonella* adhesion, attachment and survival between Ataulfo and Tommy Atkins. Therefore, only data on *Salmonella* attachment and survival employing Tommy Atkins are presented here.

### Adhesion and Attachment of *Salmonella* on Mango Surface

**FIGURE 1:**
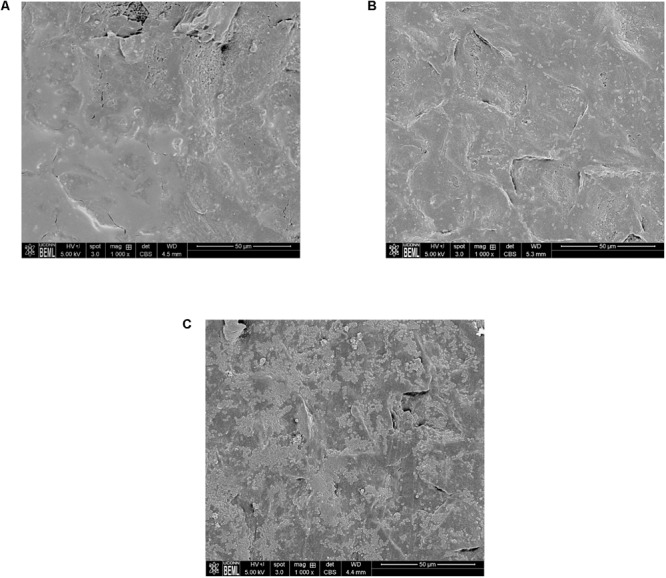
Representative SEM images of **(A)** uninoculated mango skin **(B)** mango skin at 30 s following inoculation with *S*. Newport **(C)** mango skin at 2 min following inoculation with *S*. Newport. Adhered *Salmonella* Newport are visible as rod shaped structures on the mango (var. Tommy Atkins) skin section.

**FIGURE 2:**
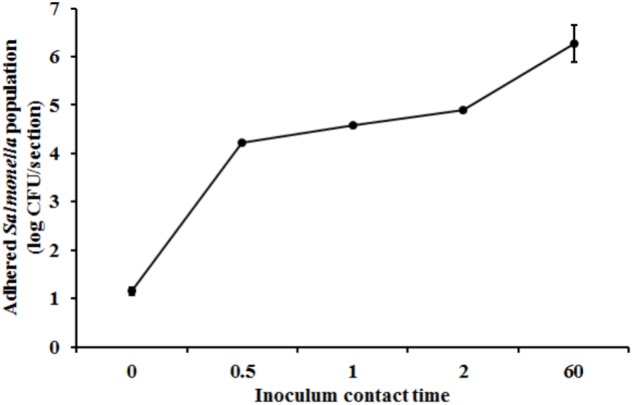
Attachment of *Salmonella* Newport on mango skin sections (var. Tommy Atkins) as a function of contact time. Data are presented as means ± SD.

**FIGURE 3:**
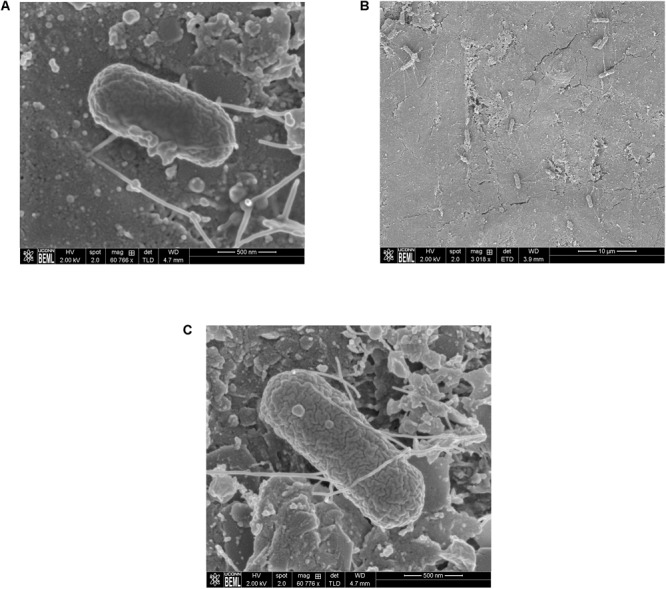
Representative SEM images showing the formation of attachment structures by *S.* Newport following adhesion to mango skin at 2 min post-inoculation **(A)** and 1 h post-inoculation **(B,C)**.

Scanning Electron Microscopy analysis of the uninoculated mango skin sections revealed the presence of a rough surface with corrugations that may favor pathogen attachment (**Figure 1A**). With SEM, the earliest contact time at which adherent *Salmonella* were observed on the mango skin was at 30 s (**Figure 1B**). However, microbiological enumeration revealed that *Salmonella* was able to adhere to the skin sections immediately on contact. As can be seen from **Figure 2**, approximately 1.2 log CFU of *Salmonella* was recovered from inoculated skin sections at the earliest sampling time. Additionally, increase in contact time was found to be associated with a significant increase in the number of adhered *Salmonella* (*p* < 0.05, **Figures 1C, 2**). For instance, between a contact time of a few second to 30 s, the number of adhered bacteria increased by ∼4 log CFU/section. Similarly, longer contact times of 1, 2 min, and 1 h were associated with a significantly higher population of adhered *Salmonella* on the mango skin sections when compared to initial contact time (**Figure 2**). These data demonstrate that within seconds of coming into contact with the frutcoplane, *Salmonella* is capable of adhering to the fruit surface. Beyond adhesion, SEM images revealed the formation of attachment structures as early as 2 min following adhesion (**Figure 3A**). Formation of extensive attachment structures that help to irreversibly anchor the pathogen on the fruit surface were observed at 1 h post-inoculation (**Figures 3B,C**). Additionally, *Salmonella* presence on the skin sections was confirmed using qPCR for the invA gene (Cheng et al., 2008). As expected, inoculated skin sections were positive for *invA* while no amplification was obtained from the control samples.

### Survival of *Salmonella* on Mangoes Under Commercial Handling and Storage Conditions

Immediately following inoculation, approximately 6.58 ± 0.04 and 4.29 ± 0.03 log CFU of *Salmonella* was recovered from the mangoes inoculated with high and low inoculum levels, respectively. After a 24 h inoculum drying period, approximately 6.52 ± 0.02 and 4.33 ± 0.06 log CFU of the pathogen were recovered from the fruits for their respective inoculation levels. These results indicate that *Salmonella* is adept at surviving on mangoes during extended drying period. Further, bacterial cells that survive on mangoes for over 24 h would probably represent populations that can withstand desiccation on the fruit surface (Lang et al., 2004). Hence all fruit were inoculated and held for 24 h prior to the survival studies.

#### Ambient Conditions and Practices Simulating the Mango Packing House

**FIGURE 4:**
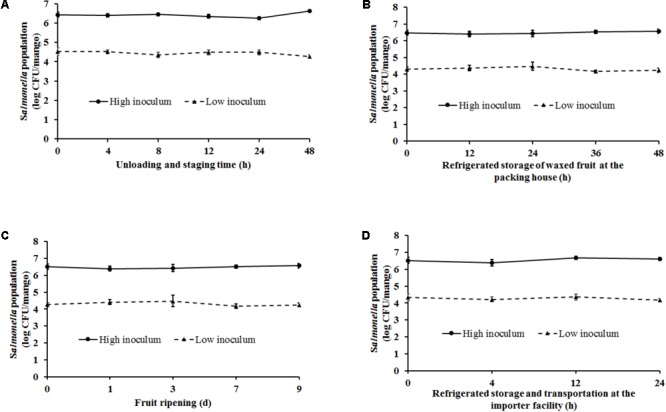
Survival of *Salmonella* on mangoes (var. Tommy Atkins) as affected by conditions simulating **(A)** fruit unloading and staging at the packing house (29–32°C, RH – 85–95%), **(B)** waxing, storage, and distribution at the packing house (10°C, RH – 85–95%), **(C)** fruit ripening at the importer facility (20–22°C, RH – 90–95%), and **(D)** post-ripening storage and distribution at the importer facility (12–15°C, RH – 90–95%). Data are presented as means ± SD.

Handling of mangoes under conditions simulating fruit unloading and staging (ambient temperature – 29–32°C, RH – 85–95%) at the packing house was found to have a significant effect on *Salmonella* populations particularly at the end of the staging period (*p* < 0.05). At high inoculum levels, *Salmonella* populations were found to increase from 6.43 ± 0.13 at 0 h to 6.61 ± 0.08 log CFU/mango at 48 h of staging (**Figure 4A**). On the other hand, inoculation of fruits with a low inoculum level was associated with a significant reduction in pathogen population by ∼0.3 log CFU/mango at the end of the experiment (**Figure 4A**).

The next stage in the process that was replicated in the lab included waxing and storage [10°C (Tommy Atkins) or 12°C (Ataulfo), RH 85–95% for 2–48 h] of mangoes. Spray application of wax by itself was not found to be associated with any reduction in pathogen population. Approximately 6.45 and 4.2 log CFU of *Salmonella* was recovered from waxed mangoes prior to storage. Following waxing fruit were stored for a period of up to 48 h to account for the time taken to move fruit from the packing house to the importer facility. As shown in **Figure 4B**, at high inoculation levels, although higher numbers of *Salmonella* were recovered from the fruits at the end of the study, data were not significantly different. Similarly, although not significantly different, mangoes inoculated with a low inoculum were associated with a decrease in *Salmonella* populations by the end of storage. For instance, *Salmonella* populations on mangoes decreased from 4.31 ± 0.13 to 4.23 ± 0.13 log CFU/mango at 0 and 48 h, respectively (**Figure 4B**).

#### Ambient Conditions and Practices Simulating the Mango Importer Facility

Once mangoes are received at the importer facility, they are stored at 20–22°C, RH 90–95% for 9 days to promote fruit ripening. Storage under these conditions was not found to significantly influence pathogen survival on fruit surface. At high and low inoculum levels, approximately 6.5 and 4.2 log CFU of *Salmonella* was recovered from the mangoes at the end of the 9 days ripening period, respectively (**Figure 4C**). Following ripening, mangoes were stored at 12–15°C, RH 90–95% for 2–24 h to simulate fruit storage and transportation from the importer facility. As previously observed, although a reduction in pathogen populations was observed at 24 h of storage specifically at low inoculum levels, these results were not found to significantly different from *Salmonella* counts at 0 h (**Figure 4D**). Overall, ambient conditions and practices simulating the packing house and importer facility were not found to significantly deter *Salmonella* survival on mangoes.

## Discussion

Although salmonellosis has been primarily associated with the consumption of foods of animal origin, of late an increasing number of fresh produce have been implicated in foodborne salmonellosis (Krtinic et al., 2010; Reddy et al., 2016). These occurrences highlight the epiphytic fitness of these enteric pathogens (Brandl, 2006). The first steps in epiphytic colonization is bacterial adhesion and attachment. In effect, the ability of a pathogen to remain attached on the fruit surface is critical to its successful colonization and survival on fresh produce (Brandl, 2006; Collignon and Korsten, 2010; Tan et al., 2016). Further, in addition to their ability to adhere, the time required to ensure adhesion and thereby prevent dislodgement may provide a survival advantage to the pathogen. In this regard results of our study demonstrate that *Salmonella* was able to adhere to the mango surface within few seconds of contact (**Figures 1B, 2**). These findings are in line with previous studies that demonstrated that *Salmonella* was able to adhere equally well on plums and peaches immediately upon contact (Collignon and Korsten, 2010).

Following the initial adhesion, non-reversible attachment of *Salmonella* on the fructoplane ensures its survival and transmission along the post-harvest continuum. As opposed to adhesion, bacterial attachment on plant surfaces is mediated by the formation of attachment structures that help to securely anchor the pathogen in place (Collignon and Korsten, 2010; Tan et al., 2016; Fornefeld et al., 2017). Toward this, results of our study demonstrate that *Salmonella* is adept at adhering and attaching to the fructoplane. As can be seen from **Figure 3A**, appearance of attachment structures can be seen as early as 2 min following initial contact. Further SEM also revealed the formation of extensive attachment structures by *Salmonella* at 1 h post-inoculation (**Figures 3B,C**). These findings are similar to previous research that demonstrated that *Salmonella* Typhimurium can attach on plums and peaches as early as 30 s and 1 h post-inoculation, respectively (Collignon and Korsten, 2010). Similarly, 6 log CFU of *S. typhimurium* was recovered from mango skin sections following an inoculum contact time of 24 h (Fernandes et al., 2014). Beyond attachment, using SEM, Gautam et al. (2014) demonstrated the presence and survival of *Salmonella* Poona on cantaloupe rinds until 24 days post-inoculation. Survival of pathogens on fresh produce, particularly in the post-harvest environment is primarily influenced by ambient fruit handling/storage conditions (temperature and relative humidity) and packing house practices such as waxing (Guo et al., 2002; Kenney and Beuchat, 2002; Shi et al., 2007; Collignon and Korsten, 2010; Huff et al., 2012; Tian et al., 2013; Zhou et al., 2014). Temperature in the packing house and fruit storage environment not only influence fruit keeping quality but also pathogen survival on fruits. Several studies have investigated the effect of different temperatures on *Salmonella* survival on fresh produce (Knudsen et al., 2001; Behrsing et al., 2003; Castro-Rosas et al., 2010; Collignon and Korsten, 2010; Huff et al., 2012; Carter et al., 2018). Knudsen et al. (2001) demonstrated that *Salmonella* could survive but not multiply on strawberries stored at 24 and 5°C for the duration of the expected shelf life. Likewise, results of our study demonstrated that irrespective of the inoculum level, *Salmonella* was able to survive but not replicate on mangoes under conditions simulating the packing house and importer facility. Similarly, Behrsing et al. (2003) investigated the ability of *Salmonella* to survive on the fruits with inedible skins including passion fruit, banana, cantaloupe, and honey dew melon. At high (5–6 log CFU/ml) and low inoculum (3 log CU/ml) levels a significant reduction in *Salmonella* populations was observed on all fruits when stored at 8°C for 7 days (cantaloupes), 10°C for 6 days (passion fruit), 12°C for 1 day (honeydew melon), and 18°C for 13 days (bananas). However, contrary to these findings, we did not observe a significant reduction in pathogen population under conditions simulating cold storage at the packing house and importer facility (**Figures 4B,D**). This could be due to fruit storage at low ambient temperature (10–12°C) but at high relative humidity (85–95%).

With specific reference to relative humidity, Tian et al. (2013) demonstrated that survival of *Salmonella* Typhimurium and *Escherichia coli* O157:H7 was significantly higher at higher relative humidity. They recovered higher populations of the pathogen from inoculated apple surface at an RH of 85% than at 65%. In fact, Tian et al. (2013) demonstrated that the survival of pathogens on fruit surface was directly proportional to RH levels with survival at RH of 100 > 85 > 68%. Similarly, Perez-Rodriguez et al. (2014) demonstrated that *Salmonella* survived on apples for 12 days when stored at 22°C and RH of 70%. Further, a recent study by Carter et al. (2018) demonstrated the survival of foodborne pathogens including *Salmonella* on packaged grapes when stored under simulated refrigerated transit conditions (1.1 ± 0.5°C; 90% RH). In corroboration with these findings, in this study, storage of fruits under high humidity and low temperatures was not found to significantly reduce *Salmonella* survival on mangoes. Approximately 6.5 log and 4 log CFU of *Salmonella* was recovered from the mangoes at the end of storage (**Figures 4B,D**).

Fruit waxing is primarily performed at the packing house to reduce moisture loss, replace natural waxes removed during washing, cover injuries, and improve the fruit’s cosmetic appearance (Crisosto et al., 1992; Kenney and Beuchat, 2002). However, Suslow et al. (2001) demonstrated that wax treatments could influence pathogen survival on stone fruits. Furthermore, they concluded that wax applied on fruit surface can provide limited dehydration protection to the pathogen and therefore favor bacterial survival. Along the same lines, Kenney and Beuchat (2002) evaluated the effect of six different wax formulations including Carnauba Gold on *S*. Muenchen survival on apples. They observed that waxing by itself did not result in any significant reduction in *Salmonella* populations on apples. Similarly, in the present study, irrespective of the inoculum level, wax application on mangoes was not associated with any reduction in *Salmonella* populations immediately and following a 2 h drying period (data not shown). Further, storage of waxed fruit at low ambient temperatures and high humidity did not impede pathogen survival on mangoes (**Figures 4B,D**).

## Conclusion

In conclusion, the results of our study demonstrate that *Salmonella* is adept at adhering attaching and surviving on mangoes under conditions simulating commercial packing house and importer facility. Further, although the currently employed time-temperature-relative humidity regimes do not promote *Salmonella* replication on mangoes, they also do not impede its survival on the produce. Hence, once contaminated, mangoes could serve as potential vehicles in the transmission of *Salmonella* along the post-harvest environment. Therefore, effective preventive measures including best management post-harvest practices are warranted to control *Salmonella* on fruits and thereby improve the microbial safety of mangoes.

## Author Contributions

MA conceived and designed the experiments. EM, MM, and DK performed the experiments. MA and MM performed the statistical analysis. EM wrote sections of the manuscript. MA wrote and revised the manuscript. All authors read and approved the submitted version.

## Conflict of Interest Statement

The authors declare that the research was conducted in the absence of any commercial or financial relationships that could be construed as a potential conflict of interest.

## References

[B1] Araiza E. Osuna T. Siller J. Contreras L. Sanchez E. (2005). Postharvest quality and shelf-life of mango cultivars grown at Sinaloa, Mexico. *Acta Hortic.* 682 1275–1281. 10.17660/ActaHortic.2005.682.170

[B2] Baskaran S. A. Upadhyay A. Kollanoor-Johny A. Upadhyaya I. Mooyottu S. Roshni Amalaradjou M. A. (2013). Efficacy of plant-derived antimicrobials as antimicrobial wash treatments for reducing enterohemorrhagic *Escherichia coli* O157:H7 on apples. *J. Food Sci.* 78 M1399–M1404. 10.1111/1750-3841.12174 24024692

[B3] Behrsing J. Jaeger J. Horlock F. Kita N. Franz P. Premier R. (2003). Survival of *Listeria innocua, Salmonella salford* and *Escherichia coli* on the surface of fruit with inedible skins. *Postharvest Biol. Technol.* 29 249–256. 10.1016/S0925-5214(03)00004-8

[B4] Beuchat L. R. Farber J. M. Garrett E. H. Harris L. J. Parish M. E. Suslow T. V. (2001). Standardization of a method to determine the efficacy of sanitizers in inactivating human pathogenic microorganisms on raw fruits and vegetables. *J. Food Prot.* 64 1079–1084. 10.4315/0362-028X-64.7.1079 11456197

[B5] Brandl M. T. (2006). Fitness of human enteric pathogens on plants and implications for food safety. *Annu. Rev. Phytopathol.* 44 367–392. 10.1146/annurev.phyto.44.070505.143359 16704355

[B6] Carter M. Q. Feng D. Chapman M. H. Gabler F. (2018). Survival of foodborne pathogens on commercially packed table grapes under simulated refrigerated transit conditions. *Food Microbiol.* 72 199–205. 10.1016/j.fm.2017.12.004 29407398

[B7] Castro-Rosas J. Santos Lopez E. M. Gomez-Aldapa C. A. Gonzalez Ramirez C. A. Villagomez-Ibarra J. R. Gordillo-Martinez A. J. (2010). Incidence and behavior of *Salmonella* and *Escherichia coli* on whole and sliced zucchini squash (*Cucurbita pepo*) fruit. *J. Food Prot.* 73 1423–1429. 10.4315/0362-028X-73.8.1423 20819351

[B8] Centers for Disease Control and Prevention [CDC] (2015). *Foodborne Illness Source Attribution Estimates for Salmonella, Escherichia coli O157 (E. coli O157), Listeria monocytogenes (Lm) and Campylobacter Using Outbreak Surveillance Data. Report from the Interagency Food Safety Analytics Collaboration (IFSAC) Project.* Available at: https://www.cdc.gov/foodsafety/pdfs/ifsac-estimating-attribution-jzk-dc-mb-508c.pdf

[B9] Centers for Disease Control and Prevention, National Center for Emerging, and Zoonotic Infectious Agents [CDC NCZEID] (2017) *National Outbreak Reporting System.* Available at: https://wwwn.cdc.gov/norsdashboard/

[B10] Cheng C.-M. Lin W. Van K. T. Phan L. Tran N. N. Farmer D. (2008). Rapid detection of *Salmonella* in foods using real-time PCR. *J. FoodProt.* 71 2436–2441. 10.4315/0362-028X-71.12.2436 19256088

[B11] Collignon S. Korsten L. (2010). Attachment and colonization by *Escherichia coli* O157:H7, *Listeria monocytogenes, Salmonella enterica* subsp. *enterica* serovar Typhimurium, and *Staphylococcus aureus* on stone fruit surfaces and survival through a simulated commercial export chain. *J. Food Prot.* 73 1247–1256. 10.4315/0362-028X-73.7.1247 20615337

[B12] Crisosto C. H. Garner D. Wiley N. Crisosto G. (1992). *Evaluation of the Effect of the Brushing and Waxing Operations on Peach and Nectarine Postharvest Performance. 1992 Annual Research Report. California Tree Fruit Agreement.* Available at: http://ucanr.edu/sites/ctfa/year/1992/?repository=51942&a=95063

[B13] Doyle M. P. Erickson M. C. (2008). Summer meeting 2007 - the problems with fresh produce: an overview. *J. Appl. Microbiol.* 105 317–330. 10.1111/j.1365-2672.2008.03746.x 18284485

[B14] Esguerra E. B. Rolle R. (2018). *Post-harvest Management of Mango for Quality and Safety Assurance. Food and Agriculture Organization of the United Nations.* Available at: http://www.fao.org/3/I8239EN/i8239en.pdf

[B15] Fatica M. K. Schneider K. R. (2011). *Salmonella* and produce: survival in the plant environment and implications in food safety. *Virulence* 2 573–579. 10.4161/viru.2.6.17880 21971184

[B16] Fernandes P. E. Sao Jose J. F. Zerdas E. R. Andrade N. J. Fernandes C. M. Silva L. D. (2014). Influence of the hydrophobicity and surface roughness of mangoes and tomatoes on the adhesion of *Salmonella enterica* serovar Typhimurium and evaluation of cleaning procedures using surfactin. *Food Control* 41 21–26. 10.1016/j.foodcont.2013.12.024

[B17] Fett W. F. (2000). Naturally occurring biofilms on alfalfa and other types of sprouts. *J. Food Prot.* 63 625–632. 10.4315/0362-028X-63.5.625 10826720

[B18] Fornefeld E. Schierstaedt J. Jechalke S. Grosch R. Smalla K. Schikora A. (2017). “Interaction between *Salmonella* and plants: potential hosts and vectors for human infection,” in *Current Topics in Salmonella and Salmonellosis* ed. Mares M. (Rijeka: InTech) 171–191.

[B19] Francis G. Gallone A. Nychas G. Sofos J. Colelli G. Amodio M. (2012). Factors affecting quality and safety of fresh-cut produce. *Crit. Rev. Food Sci. Nutr.* 52 595–610. 10.1080/10408398.2010.503685 22530712

[B20] Garrett T. R. Bhakoo M. Zhang Z. (2008). Bacterial adhesion and biofilms on surfaces. *Prog. Nat. Sci.* 18 1049–1056. 10.1016/j.pnsc.2008.04.001

[B21] Gautam D. Dobhal S. Payton M. E. Fletcher J. Ma L. M. (2014). Surface survival and internalization of *Salmonella* through natural cracks on developing cantaloupe fruits, alone or in the presence of the melon wilt pathogen *Erwinia tracheiphila*. *PLoS One* 9:e105248. 10.1371/journal.pone.0105248 25147942 PMC4141780

[B22] Goodburn C. Wallace C. A. (2013). The microbiological efficacy of decontamination methodologies for fresh produce: a review. *Food Control* 32 418–427. 10.1016/j.foodcont.2012.12.012

[B23] Guo X. Chen J. Brackett R. E. Beuchat L. R. (2002). Survival of *Salmonella* on tomatoes stored at high relative humidity, in soil, and on tomatoes in contact with soil. *J. Food Prot.* 65 274–279. 10.4315/0362-028X-65.2.274 11848557

[B24] Hanning I. B. Nutt J. D. Ricke S. C. (2009). Salmonellosis outbreaks in the United States due to fresh produce: sources and potential intervention measures. *Foodborne Pathog. Dis.* 6 635–648. 10.1089/fpd.2008.0232 19580447

[B25] Harris L. J. Beuchat L. R. Kajs T. M. Ward T. E. Taylor C. H. (2001). Efficacy and reproducibility of a produce wash in killing *Salmonella* on the surface of tomatoes assessed with a proposed standard method for produce sanitizers. *J. Food Prot.* 64 1477–1482. 10.4315/0362-028X-64.10.1477 11601693

[B26] Huff K. Boyer R. Denbow C. O’Keefe S. Williams R. (2012). Effect of storage temperature on survival and growth of foodborne pathogens on whole, damaged, and internally inoculated jalapenos (*Capsicum annuum* var. *annuum*). *J. Food Prot.* 75 382–388. 10.4315/0362-028X.JFP-11-304 22289602

[B27] Kenney S. J. Beuchat L. R. (2002). Survival of *Escherichia coli* O157:H7 and *Salmonella* Muenchen on apples as affected by application of commercial fruit waxes. *Int. J. Food Microbiol.* 77 223–231. 10.1016/S0168-1605(02)00113-7 12160082

[B28] Knudsen D. M. Yamamoto S. A. Harris L. J. (2001). Survival of *Salmonella* spp. *and Escherichia coli* O157:H7 on fresh and frozen strawberries. *J. Food Prot.* 64 1483–1488. 10.4315/0362-028X-64.10.1483 11601694

[B29] Krtinic G. Duric P. Ilic S. (2010). Salmonellae in food stuffs of plant origin and their implications on human health. *Eur. J. Clin. Microbiol. Infect. Dis.* 29 1321–1325. 10.1007/s10096-010-1001-4 20582445

[B30] Lang M. M. Harris L. J. Beuchat L. R. (2004). Evaluation of inoculation method and inoculum drying time for their effects on survival and efficiency of recovery of *Escherichia coli* O157:H7, *Salmonella*, and *Listeria monocytogenes* inoculated on the surface of tomatoes. *J. Food Prot.* 67 732–741. 10.4315/0362-028X-67.4.732 15083725

[B31] Luo Y. Nou X. Millner P. Zhou B. Shen C. Yang Y. (2012). A pilot plant scale evaluation of a new process aid for enhancing chlorine efficacy against pathogen survival and cross-contamination during produce wash. *Int. J. Food Microbiol.* 158 133–139. 10.1016/j.ijfoodmicro.2012.07.008 22857846

[B32] Marshall K. C. (1992). Biofilms: an overview of bacterial adhesion, activity, and control at surfaces. *Am. Soc. Microbiol. News* 58 202–208.

[B33] Mathew E. N. Muyyarikkandy M. S. Bedell C. Amalaradjou M. A. (2018). Efficacy of chlorine, chlorine dioxide and peroxyacetic acid in reducing *Salmonella* contamination in wash water and on mangoes under simulated mango packinghouse washing operations. *Front. Sustain. Food Syst.* 2:18 10.3389/fsufs.2018.00018

[B34] Monier J. M. Lindow S. E. (2005). Aggregates of resident bacteria facilitate survival of immigrant bacteria on leaf surfaces. *Microb. Ecol.* 49 343–352. 10.1007/s00248-004-0007-9 16003469

[B35] Morris C. E. Monier J. M. (2003). The ecological significance of biofilm formation by plant-associated bacteria. *Annu. Rev. Phytopathol.* 41 429–453. 10.1146/annurev.phyto.41.022103.134521 12730399

[B36] National Mango Board [NMB] (2014b). *Mango Postharvest Best Management Practices Manual.* Available at: http://www.mango.org/sites/default/files/download/mango_manual.pdf

[B37] National Mango Board [NMB] (2014a). *Mango Handling and Ripening Protocol.* Available at: http://www.mango.org/sites/default/files/Mango_Handling_and_Ripening_Protocol.pdf

[B38] Parnell T. L. Harris L. J. (2003). Reducing *Salmonella* on apples with wash practices commonly used by consumers. *J. Food Prot.* 66 741–747. 10.4315/0362-028X-66.5.741 12747679

[B39] Parnell T. L. Harris L. J. Suslow T. V. (2005). Reducing *Salmonella* on cantaloupes and honeydew melons using wash practices applicable to postharvest handling, foodservice, and consumer preparation. *Int. J. Food Microbiol.* 99 59–70. 10.1016/j.ijfoodmicro.2004.07.014 15718029

[B40] Penteado A. L. Eblen B. S. Miller A. J. (2004). Evidence of *Salmonella* internalization into fresh mangos during simulated postharvest insect disinfestation procedures. *J. Food Prot.* 67 181–184. 10.4315/0362-028X-67.1.181 14717371

[B41] Penteado A. L. (2017). Microbiological safety aspects of mangoes (*Mangifera indica*) and papayas (*Carica papaya*): a mini-review. *Vigil. Sanit. Debate* 5 127–140. 10.22239/2317-269x.00779

[B42] Perez-Rodriguez F. Begum M. Johannessen G. (2014). Study of the cross-contamination and survival of *Salmonella* in fresh apples. *Int. J. Food Microbiol.* 184 92–97. 10.1016/j.ijfoodmicro.2014.03.026 24774564

[B43] Reddy S. P. Wang H. Adams J. K. Feng P. C. (2016). Prevalence and characteristics of *Salmonella* serotypes isolated from fresh produce marketed in the United States. *J. Food Prot.* 79 6–16. 10.4315/0362-028X.JFP-15-274 26735024

[B44] Sheng L. Edwards K. Tsai H. C. Hanrahan I. Zhu M. J. (2017). Fate of *Listeria monocytogenes* on fresh apples under different storage temperatures. *Front. Microbiol.* 8:1396. 10.3389/fmicb.2017.01396 28790993 PMC5522875

[B45] Shi X. Namvar A. Kostrzynska M. Hora R. Warriner K. (2007). Persistence and growth of different *Salmonella* serovars on pre– and postharvest tomatoes. *J. Food Prot.* 70 2725–2731. 10.4315/0362-028X-70.12.2725 18095423

[B46] Sivapalasingam S. Barrett E. Kimura A. Van Duyne S. De Witt W. Ying M. (2003). A multistate outbreak of *Salmonella enterica* serotype newport infection linked to mango consumption: impact of water-dip disinfestation technology. *Clin. Infect. Dis.* 37 1585–1590. 10.1086/379710 14689335

[B47] Strawn L. K. Danyluk M. D. (2010). Fate of *Escherichia coli* O157:H7 and *Salmonella* spp. on fresh and frozen cut mangoes and papayas. *Int. J. Food Microbiol.* 138 78–84. 10.1016/j.ijfoodmicro.2009.12.002 20022397

[B48] Suslow T. George A. Fernandez L. Cifuentes R. (2001). *Assessment of Production and Retail Handling Practices of Peaches, Plums and Nectarines on Microbial Food Safety Risk Reduction. 2001 Annual Research Report. California Tree Fruit Agreement.* Available at: http://ucanr.edu/sites/ctfa/category/Food_Safety/?repository=46433&a=95235

[B49] Tan M. S. Moore S. C. Tabor R. F. Fegan N. Rahman S. Dykes G. A. (2016). Attachment of *Salmonella* strains to a plant cell wall model is modulated by surface characteristics and not by specific carbohydrate interactions. *BMC Microbiol.* 16:212. 10.1186/s12866-016-0832-2 27629769 PMC5024418

[B50] Tian J. Q. Bae Y. M. Lee S. Y. (2013). Survival of foodborne pathogens at different relative humidities and temperatures and the effect of sanitizers on apples with different surface conditions. *Food Microbiol.* 35 21–26. 10.1016/j.fm.2013.02.004 23628610

[B51] Tomas-Callejas A. Lopez-Velasco G. Camacho A. B. Artes F. Artes-Hernandez F. Suslow T. V. (2011). Survival and distribution of *Escherichia coli* on diverse fresh-cut baby leafy greens under preharvest through postharvest conditions. *Int. J. Food Microbiol.* 151 216–222. 10.1016/j.ijfoodmicro.2011.08.027 21924789

[B52] Wei C. Huang T. Kim J. Lin W. Tamplin M. Bartz J. (1995). Growth and survival of *Salmonella* Montevideo on tomatoes and disinfection with chlorinated water. *J. Food Prot.* 58 829–836. 10.4315/0362-028X-58.8.829 31137395

[B53] Zhou B. Luo Y. Nou X. Yang Y. Wu Y. Wang Q. (2014). Effects of postharvest handling conditions on internalization and growth of *Salmonella enterica* in tomatoes. *J. Food Prot.* 77 365–370. 10.4315/0362-028X.JFP-13-307 24674426

